# Correlation Between the Functional Connectivity of Basal Forebrain Subregions and Vigilance Dysfunction in Temporal Lobe Epilepsy With and Without Focal to Bilateral Tonic-Clonic Seizure

**DOI:** 10.3389/fpsyt.2022.888150

**Published:** 2022-06-02

**Authors:** Binglin Fan, Linlin Pang, Siyi Li, Xia Zhou, Zongxia Lv, Zexiang Chen, Jinou Zheng

**Affiliations:** ^1^Department of Neurology, The First Affiliated Hospital of Guangxi Medical University, Nanning, China; ^2^Department of Neurology, The People's Hospital of Guangxi Zhuang Autonomous Region, Nanning, China

**Keywords:** temporal lobe epilepsy, focal to bilateral tonic-clonic seizure, basal forebrain subregions, functional connectivity, vigilance function

## Abstract

**Purpose:**

Previous research has shown that subcortical brain regions are related to vigilance in temporal lobe epilepsy (TLE). However, it is unknown whether alterations in the function and structure of basal forebrain (BF) subregions are associated with vigilance impairment in distinct kinds of TLE. We aimed to investigate changes in the structure and function BF subregions in TLE patients with and without focal to bilateral tonic-clonic seizures (FBTCS) and associated clinical features.

**Methods:**

A total of 50 TLE patients (25 without and 25 with FBTCS) and 25 healthy controls (HCs) were enrolled in this study. The structural and functional alterations of BF subregions in TLE were investigated using voxel-based morphometry (VBM) and resting-state functional connectivity (rsFC) analysis. Correlation analyses were utilized to investigate correlations between substantially altered imaging characteristics and clinical data from patients.

**Results:**

FBTCS patients had a lower rsFC between Ch1-3 and the bilateral striatum as well as the left cerebellum posterior lobe than non-FBTCS patients. In comparison to non-FBTCS patients, the rsFC between Ch4 and the bilateral amygdala was also lower in FBTCS patients. Compared to HCs, the TLE patients had reduced rsFC between the BF subregions and the cerebellum, striatum, default mode network, frontal lobe, and occipital lobes. In the FBTCS group, the rsFC between the left Ch1-3 and striatum was positive correlated with the vigilance measures. In the non-FBTCS group, the rsFC between the left Ch4 and striatum was significantly negative correlated with the alertness measure.

**Conclusion:**

These results extend current understanding of the pathophysiology of impaired vigilance in TLE and imply that the BF subregions may serve as critical nodes for developing and categorizing TLE biomarkers.

## Introduction

Temporal lobe epilepsy (TLE), the most frequent form of focal epilepsy in humans, is characterized by sclerosis of the medial temporal lobe and recurring seizures that mainly occur in the hippocampus and amygdala (1). In general, TLE can be classified into three categories: focal awareness seizures (FAS), focal impaired awareness seizures (FIAS), and focal to bilateral tonic-clonic seizures (FBTCS) (2). Over one-third of patients with TLE suffer from FBTCS, which can result in injury or death as a result of accidents or falls, as well as seizure-related brain damage and, in severe or prolonged cases, sudden death (3, 4).

Patients with TLE frequently experience a variety of cognitive, mental, and behavioral impairments, most notably affecting memory, executive function, language, and attention, making it difficult to perform routine tasks, work, and maintain personal relationships, all of which have a negative effect on their quality of life (5, 6). While many factors can contribute to or exacerbate cognitive impairment, the type of seizure has a substantial impact on TLE patients' cognitive prognosis (7). Numerous FBTCS patients suffer from severe cognitive impairments, the most common of which are difficulties with attention and memory (8). Attention is the cornerstone of all cognitive function, and alertness is the most crucial component of attention (9). FBTCS is the most severe form of TLE and is associated with significantly more cognitive impairment than other forms of TLE. Thus, it is critical to shed light on impaired alertness in FBTCS patients.

Cognitive deficits in TLE patients and animal models of limbic seizures have been linked to anomalies in subcortical brain areas that regulate vigilance (10, 11). Subcortical structures regulate vigilance by modifying sleep-wake rhythms and consciousness, and their impairment may result in sleep-wake abnormalities, impaired vigilance, and even consciousness disturbance (12). The main subcortical structures are the ascending reticular activating system (ARAS) nuclei in the brainstem, basal forebrain (BF), intralaminar thalamic nuclei, pulvinar, and posterior hypothalamus (13). The locus coeruleus (LC), a component of the ARAS nuclei, and the thalamus are two subcortical brain areas that are involved in alertness and attention (8, 14). Converging evidence indicates that anatomical and functional anomalies in the basal forebrain can result in insomnia or parasomnias (15–17). Furthermore, according to Adaptive Resonance Theory, Stephen Grossberg proposed that acetylcholine (ACh) release by cells in the basal forebrain can modulate vigilance (18). However, whether the basal forebrain influences alertness in TLE patients is unclear.

The BF, which consists of four subcellular groups (Ch1–4), is critical for the generation and distribution of ACh to the neocortex, amygdala, and hippocampus (19, 20), as well as for modulating neuron excitability and numerous cognitive functions (21). Recent studies have proven that significant BF neuron degeneration and loss of cortical cholinergic innervation promote cognitive decline in Alzheimer's disease, Parkinson's disease, Wilson's disease, and multiple sclerosis (22–25). Hern'an et al. observed aberrant functional connectivity and community between the nucleus basalis of Meynert (NBM) and cerebral hemisphere, related to cognitive impairments in TLE(1).

We postulate that BF neurons deteriorate in patients with different kinds of TLE, disrupting their innervated functional networks and resulting in alert impairment. To verify this hypothesis, we examined the gray volume of the BF in TLE patients with and without FBTCS as well as healthy controls (HCs), followed by functional connectivity analysis to identify aberrant connectivity between the BF subregions (Ch1-4) and the cerebral hemisphere. Linear regression was used to determine whether abnormalities in the structure and functional connectivity in patients with TLE were linked to their clinical data.

## Methods

### Participants

Patients with unilateral TLE were enrolled from the Department of Neurology at the First Affiliated Hospital of Guangxi Medical University between January 2017 and September 2021. According to the classification standard guidelines of 1981, 1989 and 2017 formulated by ILAE (26), the secondary generalization diagnosis of complex partial seizures and partial seizures was performed. Inclusion criteria were as follows: (1) all patients had unilateral (left and right) TLE, which was confirmed by MRI structural image, video electroencephalography (EEG) assessment and clinical manifestation analysis. (2) All patients took antiepileptic drugs (AEDs) regularly; (3) All patients were right-handed. Exclusion criteria were as follows: (1) comorbidities affecting cognitive function, including traumatic brain injury, intracranial tumor, stroke, infection, multiple sclerosis, and Alzheimer's disease; (2) Patients with a score <24 on MMSE; (3) patients who take or are taking topiramate and barbiturates; (4) MRI structural images showing abnormalities except hippocampal sclerosis.

Every patient had FIAS and/or FAS, with some also having FBTCS. Patients were divided into two groups for this study based on their medical history at the time of scanning. The “non-FBTCS” group included 25 patients who had never experienced FBTCS, the “FBTCS” group included 25 patients with recurrent FBTCS in the year before scanning, and 25 healthy control subjects with matching demographic features were recruited as a neuroimaging reference group. As confirmed by health screening techniques, the control group had no psychological or neurological issues. All procedures were approved by the Ethics Committee of Guangxi Medical University's First Affiliated Hospital. All individuals provided written informed consent for the study.

### Neuropsychological Assessment

The Montreal Cognitive Assessment (MoCA) was used to evaluate cognitive impairment.

The ANT was used to assess each subject's vigilance, as previously stated (27). Participants were instructed to keep their eyes on a fixation cross in the screen's center and determine the direction of the target arrow throughout the trials. Participants were told to press a button to provide an answer as accurately and quickly as they could. The formal test included three blocks of 96 trials, plus the practice block. The entire test took approximately 25 min. In the test, participants had to decide whether the middle arrow would point left or right next. They were given three types of hints: (1) a center cue, characterized by the presence of an asterisk at the central fixation point; (2) double cues, characterized by an asterisk positioned above and below the fixation cross; or (3) no cue, characterized by a pair of arrows pointing in the opposite direction as the target arrow, or a pair of dashes, flanking the fixation point. Each test contained unique clues, targets, and surrounding interference data, and they were presented in a random order. The device automatically detected and recorded participants' reaction time (RT). the no-cue condition indicated tonic alertness and represented a state of general wakefulness, similar to sustained attention. The double-cue condition indicated phasic alertness and represented the ability of the participant to be response ready for a short period of time subsequent to the presentation of external cues or stimuli. Alertness was reflected by the RT in the two different warning conditions. Alertness was determined by subtracting the median of the double cue condition from the median of the no cue condition. The larger the alertness value was, the greater the degree of alertness.

### Imaging Acquisition

The images of the participants were acquired at the First Affiliated Hospital of Guangxi Medical University utilizing a 3.0 Tesla MRI scanner (Philips, The Netherlands) equipped with a standard eight-channel head coil. Throughout the scan, all individuals were instructed to close their eyes and relax but not to sleep. Foam cushions and noise-canceling earplugs were used to reduce noise and head movements. A T1-3D BRAVO sequence was used to acquire high-resolution sagittal T1WI images with the following acquisition parameters: repetition time (TR) = 7.8 ms, echo time (TE) = 3.4 ms, flip angle = 9°, field of view (FOV) = 256 × 256 mm, matrix = 256 × 256 mm, slice thickness = 1 mm without slice gap, voxel size = 0.89 × 0.89 × 1 mm, and 176 sagittal slices. Resting-state functional MRI images were collected by using gradient-echo single-shot echo-planar imaging sequences with a TR = 2,000 ms, TE = 30 ms, FOV = 220 × 220 mm, FA = 90°, matrix = 64 × 64, slice thickness = 3.5 mm, slice gap = 0.5 mm, voxel size = 3.44 × 3.44 × 4 mm, 41 slices, and 225 volumes. For data quality control, the scan was evaluated by two professional neuroradiologists who were blinded to the clinical information.

### MRI Analysis

#### Resting-State fMRI Data Pre-processing

All resting-state functional MRI images were preprocessed using the data processing and analysis for brain imaging (DPABI) software (http://rfmri.org/dpabi), which is based on SPM12 and runs on MATLAB R2018b. First, the first ten volumes of each participant were discarded, and slice timing correction was used to account for the temporal delay between slices. By realigning all functional images to the center image, we excluded subjects who moved their heads more than 2 mm or 2°. Then, the motion-corrected functional volumes were coregistered with the high-resolution anatomical images and standardized to the MNI space. Space smoothing was performed using a 6-mm full-width at half maximum (FWHM) Gaussian kernel. Low-frequency drift and high-frequency noise were reduced by detrending the data. Finally, the covariance of head movement, mean white matter signal and cerebrospinal fluid were regressed, and the residual signals were filtered at 0.08–0.1 Hz.

#### Structural MRI Data Pre-processing

We used the cat12 toolbox (http://www.neuro.unijena.de/cat/), which is based on the SPM12 package (http://www.fil.ion.ucl.ac.uk/spm/software/spm12/), to process structural images. First, we used an adaptive maximum a posteriori technique to segment individual structural images into gray matter, white matter, and CSF. Next, the generated gray matter maps were normalized to MNI space using a high-dimensional “DARTEL” technique and then adjusted for spatial normalization effects. Finally, the gray matter maps were smoothed spatially using an 8-mm FWHM Gaussian kernel.

#### Definition of BF Subregions

Subregions of the BF were defined utilizing stereotaxic probabilistic maps of the BF's cytoarchitectonic boundaries generated by the SPM Anatomy Toolbox (13). Ch1-4 were defined using the Anatomy toolkit's BF probability maps. Following a 50% probability threshold, the ROIs were resampled and warped to the MNI space.

#### Gray Matter Volume of BF Subregions

The mean gray matter volume (GMV) of each subject was computed across all voxels, and each BF subregion was subsequently evaluated. The volume of the BF was determined using CAT12, which is based on SPM12. They were then non-linearly registered to the MNI152 standard space after segmentation. Each study's GM template was created by averaging and flipping the normalized images. To account for the non-linear component of the transformation, all native GM images were divided by the warp field's Jacobian. Finally, an isotropic 3-mm Gaussian kernel was used to smooth the modulated GM images. A BF mask was used to extract each participant's BF volume. The collected BF volumes were also analyzed statistically.

### Functional Connectivity Analysis

#### Seed-Based Resting-State Functional Connectivity of BF Subregions

The mean time series for each BF subregion was acquired first and then correlated with the time series for each voxel throughout the entire brain (Pearson correlation). As a result, each subject's whole-brain resting-state functional connectivity (rsFC) was provided in four maps. To normalize the rsFC maps, Fisher's r-to-z transformation was used on each of the generated maps.

### Statistical Analysis

SPSS 20.0 (SPSS Inc., Chicago, IL, USA) was used to perform statistical analyses. To compare normally distributed data among the three groups (*P* < 0.05), one-way analysis of variance (ANOVA) was employed, and chi-square tests were utilized to analyze categorical variables. To compare clinical factors between the two groups of patients, Student's *t*-tests for normally distributed variables and Mann–Whitney tests for non-normally distributed data were used. P < 0.05 was chosen as the level of statistical significance.

We used DPABI's statistical analysis toolkits to compare FC and GMV maps for each ROI among the three groups. ANOVA was used to compare the imaging variables among the groups, with age, sex, and head motion as covariates (P < 0.05). Multiple comparisons were corrected using a false discovery rate (FDR) correction for clusters with more than 30 voxels. Then, pairwise comparisons of regions with significant group differences in FC were undertaken. We used two-tailed paired comparison *t*-tests and a false discovery rate (FDR) correction at *P* < 0.05. Correlation analyses between imaging characteristics and clinical factors for patients were conducted in SPSS 20.0, revealing substantial group differences. Multiple comparisons were adjusted using the Bonferroni method. Parametric comparisons were performed with Pearson's correlation analysis, and non-parametric comparisons were performed with Spearman's correlation analysis.

### Multiclass Discriminant Analysis

To ascertain the ability of seed-based rsFC to discriminate among the three groups, multiclass discriminant analysis was performed using PRoNTo v2.0 software in MATLAB 2018b (28). Specifically, the multiclass classification was transformed into three binary classifiers using a one-vs.-one coding method. To reduce the dimensionality of initial features, starting features were selected from voxels with significant group effects (*P* < 0.05, uncorrected). After that, the outputs of all binary classifiers were combined using an error-correcting output code technique. A 10-fold cross-validation approach was used to assure generalization during this process. Finally, we calculated the total and group-specific accuracies.

## Results

### Clinical and Demographic Characteristics

The demographic and clinical data of all participants are summarized in Table 1. No differences in age, sex, handedness, seizure focus, age of seizure onset, disease duration, or seizure frequency were discovered among the three groups (*P* > 0.05). However, the RTs on the no cue and double cue conditions were significantly different among these three groups. A *post-hoc* test revealed no significant differences between FBTCS and non-FBTCS patients but significant differences between FBTCS or non-FBTCS patients and HCs. In addition, the RTs of the TLE group were longer than those of the HC group.

**Table 1:** The demographic and clinical characteristics of the two TLE groups and HCs.

	**FBTCS group (25)**	**Non-FBTCS group (25)**	**HCs (25)**	***P*-value**
Age (M ± SD)	31.28 ± 8.44	32.72 ± 11.99	27.16 ± 5.88	0.091^b^
Sex (M/F)	9/16	5/20	10/15	0.276^a^
Handedness, R/L/A	24/1/0	23/1/1	22/2/1	0.982^*^
Seizure focus, LT/RT	12/13	11/14	NA	0.78^a^
Age of seizure onset, years (M ± SD)	24.28 ± 7.14	23.86 ± 8.93	NA	0.427^e^
Duration of disease, years, median (range)	7.2 (2.5–35.3)	7.6 (3.0–21.0)	NA	0.319^d^
Frequency of seizure, n/month, median (range)	2.0 (0–12.0)	2.0 (0–10.0)	NA	0.99^d^
**Seizure type**				
FAS	0	5		
FIAS	0	17		
FAS + FIAS		3		
FAS + FBTCS	5	0		
FIAS + FBTCS	20	0		
Mean FD, mm (mean ± SD)	0.057 ± 0.030	0.070 ± 0.053	0.049 ± 0.021	0.143^c^
**Current antiepileptic drugs by category**				
VGNC	18	16		
GABAa agonist	4	2		
SV2a receptor-mediated	12	8		
CRMP2 receptor-mediated	3	1		
Multiaction	7	5		
MoCA (mean ± SD)	26.92 ± 2.86	26.32 ± 3.28	28.80 ± 1.58	0.005^b^^*^
RT_no−cue_ (ms)	694.32 ± 140.58	716.12 ± 97.92	601.10 ± 68.36	0.001^bΔ^
RT_double−cue_ (ms)	650.05 ± 145.30	650.06 ± 100.01	554.35 ± 60.24	0.003^b^†^^
Alertness (ms)	0.082 ± 0.040	0.081 ± 0.046	0.084 ± 0.033	0.188^b^

a *P was calculated using the chi-square test*;

b *P was calculated using an ANOVA*;

c *P was calculated using the Kruskal–Wallis test*;

d *P was calculated using the Mann–Whitney test*;

e *P was calculated using two independent sample t-tests; Fisher's exact test was performed instead, as 20% of cells had an expected count <5*.

* *post-hoc comparison showed a significant difference between FBTCS and non-FBTCS patients and HCs, no difference between FBTCS and non-FBTCS patients; Δ, post-hoc comparison showed a significant difference between FBTCS and non-FBTCS patients and HCs, no difference between FBTCS and non-FBTCS patients*;

† *post-hoc comparison showed a significant difference between FBTCS and non-FBTCS patients and HCs, no difference between FBTCS and non-FBTCS patients; HCs, healthy controls; M, male; F, female; M ± SD, mean ± standard deviation; FAS, focal aware seizures; FIAS, focal impaired awareness seizures; FBTCS, focal to bilateral tonic–clonic seizures; FD, framewise displacement; AEDs, antiepileptic drugs; VGNC, voltage-gated Na^+^ channel blockers, e.g., oxcarbazepine, lamotrigine (plus T Type Ca2^+^ channel blockers); SV2a receptor mediated, e.g., levetiracetam; Multiaction, e.g., Na^+^ valproate (VGNC + GABAa agonist), topiramate (VGNC + GABAa agonist + AMPA/kainate receptor blocker + carbonic anhydrase inhibitor). Multiple antiepileptic drugs in the same category taken by one patient were only counted once. ANOVA, one-way analysis of variance; χ^2^, chi-square tests; NA, not available; MoCA, Montreal Cognitive Assessment; RT, response time; FBTCS, focal to bilateral tonic-clonic seizures; and non-FBTCS, no focal to bilateral tonic-clonic seizures*.

### Intact GMV in the Patients

There was no significant difference in GMV among the three groups for the BF subregions (*P* > 0.05 [FDR corrected]).

### rsFC Values Differed Between Groups

**Figure 1:**
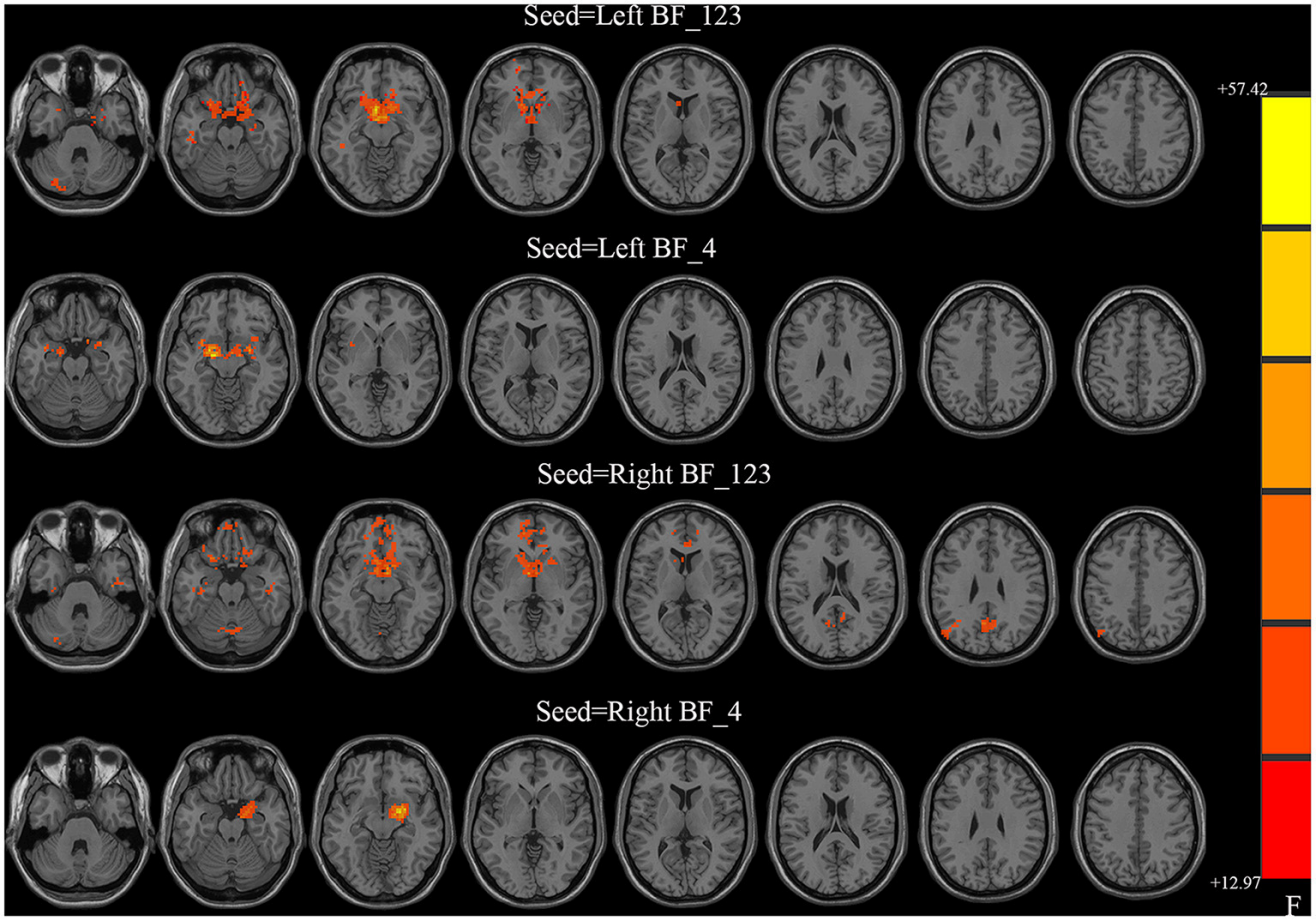
Between-group differences in the resting-state functional connectivity (RSFC) with all four basal forebrain subregions in groups with temporal lobe epilepsy (TLE). The colored bars indicate the *P* values. SPM software was used to map the data onto the brain's surface.

All four seeds exhibited significant group effects (Figure 1 and Table 2). Specifically, significant alterations in rsFC were detected in four clusters in the left Ch1-3, including rsFC with the right cerebellar posterior lobe, bilateral striatum, left superior frontal gyrus, and right middle temporal gyrus. Significant alterations in rsFC were found in four clusters of the right Ch1-3, primarily rsFC with the right cerebellar posterior lobe, bilateral striatum, right precuneus, and left middle occipital gyrus. The bilateral amygdala had significant anomalies in rsFC with the left Ch4, and the left amygdala had significant anomalies in rsFC with the right Ch4.

**Table 2:** Group differences in FC.

**ROI**	**Cluster**	**Brain regions/AAL**	**Peak MNI coordinates**			**Cluster size**	**Peak F value**
			**X**	**Y**	**Z**		
LBF_123	Cluster 1	Cerebellum Posterior Lobe_L	−27	−84	−36	44	13.3895
	Cluster 2	Striatum_L/Striatum_R	−6	9	−12	1066	47.6569
	Cluster 3	Superior Frontal Gyrus_L	−21	66	−6	35	12.9727
	Cluster 4	Temporal_Mid_L	−45	−33	−15	30	13.7519
LBF_4	Cluster 1	Amygdala_R	27	−3	−12	39	19.2485
	Cluster 2	Amygdala_L	−18	−9	−12	272	38.1336
RBF_123	Cluster 1	Cerebellum Posterior Lobe_L	−21	−87	−36	39	13.4447
	Cluster 2	Striatum_L/Striatum_R	6	0	−9	808	41.9343
	Cluster 3	Precuneus_R	3	−57	24	124	14.2744
	Cluster 4	Occipital_Mid_L	−48	−78	33	54	15.4561
RBF_4	Cluster 1	Amygdala_R	21	−3	−12	235	57.4224

*The results were corrected by FDR. ROI, regions of interest; AAL, automated anatomical labeling atlas; MNI, Montreal Neurological Institute*.

**Figure 2:**
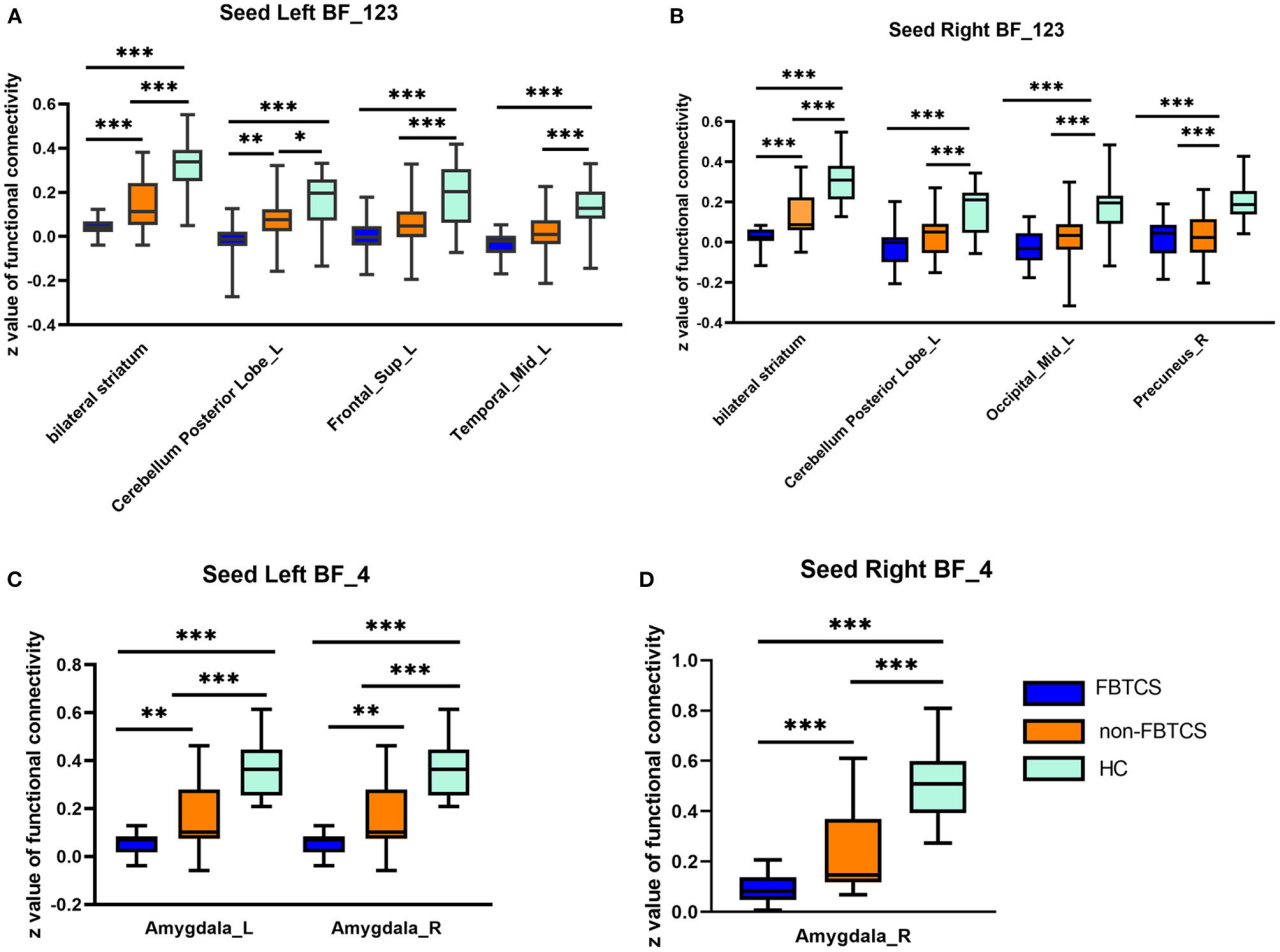
**(A–D)** Altered resting-state functional connectivity (RSFC) of the basal forebrain (BF) subregions in patients with temporal lobe epilepsy (TLE). HCs, healthy controls. ***P < 0.001; **P < 0.01; *P < 0.05.

With the left Ch1-3 as a seed, the FBTCS and non-FBTCS patients showed considerably lower FC than HCs with the right cerebellum posterior lobe, bilateral striatum, left superior frontal gyrus, and right middle temporal gyrus, respectively, and the FBTCS group demonstrated decreased FC compared to HCs with the right cerebellum posterior lobe, bilateral striatum, and left superior frontal gyrus, respectively (Figure 2A). Using the right Ch1-3 as a seed, the FBTCS and non-FBTCS groups showed significantly decreased FC compared to HCs with the right cerebellar posterior lobe, bilateral striatum, right precuneus, and left middle occipital gyrus, respectively, and the FBTCS group showed lower FC than the non-FBTCS group with the bilateral striatum (Figure 2B). When the left Ch4 was used as a seed, the FBTCS group showed significantly lower FC than the non-FBTCS and HC groups with the bilateral amygdala (Figure 2C). Using the right Ch4 as a seed, the FBTCS group showed significantly decreased FC with the right amygdala compared with the non-FBTCS and HC groups (Figure 2D).

To further explore whether there were differences in the brain regions between left and right mTLE in the different TLE groups, an ANOVA was performed. We found that there was no significant difference (*P* > 0.05) between the two TLE groups. Details are shown in Supplementary Material 1.

### Correlations Between Altered rsFC With the BF Subregions and Clinical Characteristics

**Figure 3:**
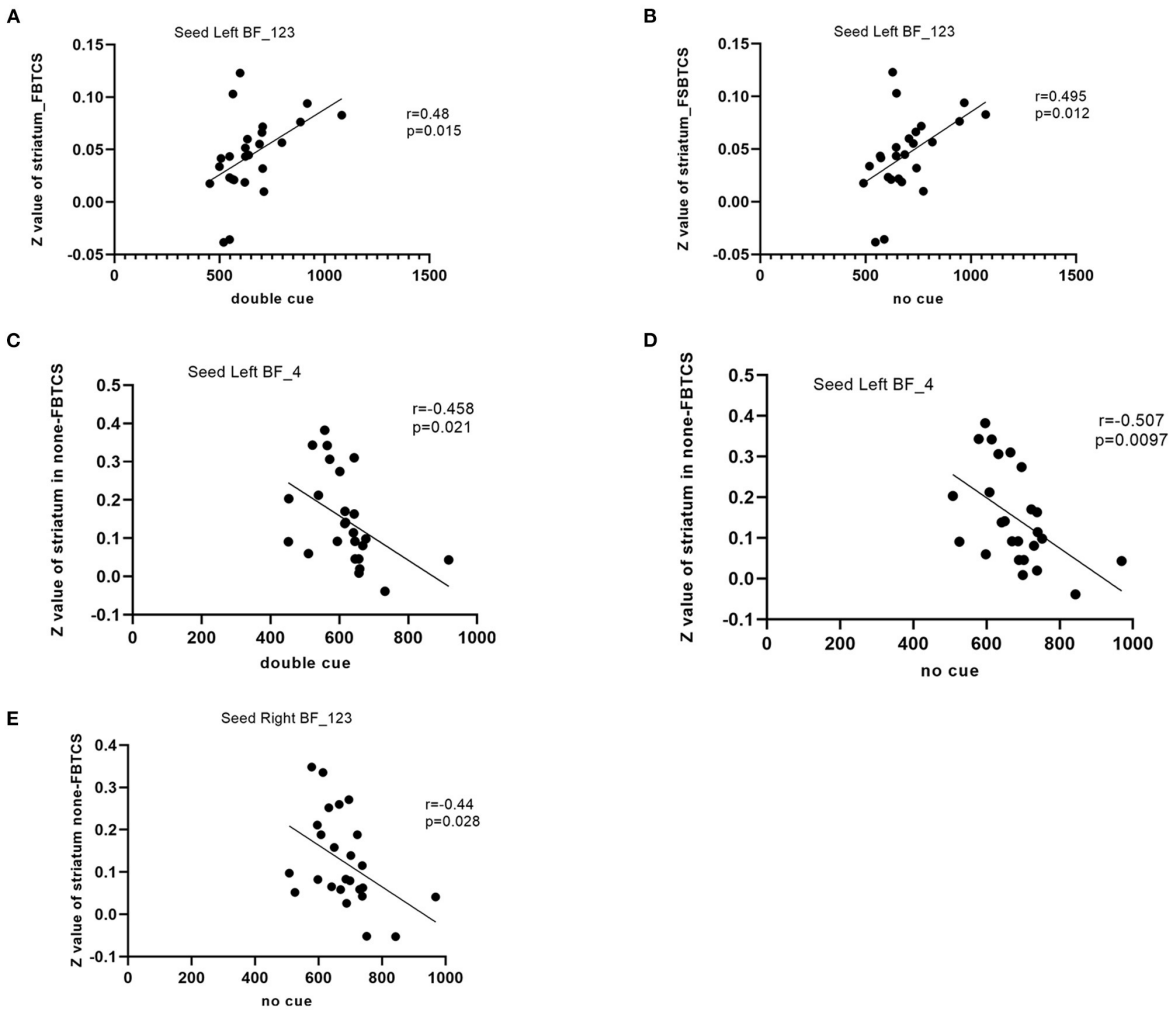
**(A–E)** Scatter plots depicting the correlations between the altered resting-state functional connectivity (RSFC) in the BF subregions and the clinical variables in patients.

The rsFC between the left Ch1-3 and striatum had a significant positive correlation with performance in the double cue (*r* = 0.48, *P* = 0.015) and no cue conditions (*r* = 0.495, *p* = 0.012) in the FBTCS group. In the non-FBTCS group, the rsFC between the left Ch4 and striatum had a moderate negative correlation with performance in the double cue (*r* = −0.458, *P* = 0.021) and no cue conditions (*r* = −0.507, *p* = 0.0097); additionally, the rsFC between the right Ch1-3 and striatum had a moderate negative correlation with performance in the no cue condition (*r* = −0.44, *p* = 0.028) (Figure 3).

### Multiclass Classification

Table 3 summarizes the overall and group-level accuracy. In general, among fourth basal forebrain subregions to voxel functional connectivity in differentiating subjects from each other, the right Ch1-3 and left Ch4 subregions performed better than the left Ch1-3 and right Ch4 subregions in identifying the three groups (accuracy 85.33 and 81.00% vs. 78.67 and 78.67%).

**Table 3:** Results of multiclass classification.

**Seed**	**Actual class**	**Predicted class**			**Accuracy (%)**	
		**HC**	**Non-FBTCS**	**FBTCS**	**Group**	**Total**
Left Ch1-3	HCs	19	4	2	76.00	78.67
	Non-FBTCS	5	18	2	72.00	
	FBTCS	3	0	22	88.00	
Right Ch1-3	HCs	22	2	1	88.00	85.33
	Non-FBTCS	3	20	2	80.00	
	FBTCS	0	2	22	88.00	
Left Ch4	HCs	20	5	0	80.00	81.00
	Non-FBTCS	3	21	1	84.00	
	FBTCS	0	5	20	80.00	
Right Ch4	HCs	21	2	2	84.00	78.67
	Non-FBTCS	4	20	1	80.00	
	FBTCS	5	2	18	72.00	

*HCs, healthy controls; FBTCS, focal to bilateral tonic-clonic seizure*.

## Discussion

In this study, we investigated the structural and functional changes in BF subregions in patients with different kinds of TLE as well as HCs by analyzing VBM and functional connectivity. Our primary findings were as follows: (i) the rsFC between Ch1-3 and the bilateral striatum as well as the left cerebellar posterior lobe was considerably lower in FBTCS patients than in non-FBTCS patients. Additionally, patients with FBTCS had reduced rsFC between Ch4 and the bilateral amygdala. In comparison to HCs, the two TLE groups showed significantly lower rsFC between the basal forebrain subregions and bilateral hemisphere, most notably in the FC between the BF subregions and the cerebellum, striatum, default mode network, frontal lobe, and occipital lobe. (ii) Significant positive or negative correlations were observed between abnormal rsFC with the striatum and alertness metrics. Overall, our findings suggest that disrupted cholinergic activity may contribute to decreased vigilance in many types of TLE, providing a more complete explanation of the cognitive mechanism underlying pathological damage.

### Changes in the BF Structure

The BF is located in the front of the forebrain, beneath the striatum. The BF has many cholinergic projections to the neocortex, which is involved in the neuromodulation of cognitive performance. In this study, there was no difference in the volume of the BF subregions among the three groups. Memory loss has been linked to Ch4 neuron deterioration in neurodegenerative disorders such as Alzheimer's disease, mild cognitive impairment, Parkinson's disease with moderate cognitive impairment, and Wilson disease (29, 30). However, Ma et al. found no difference in the volume of BF subregions in a previous study on short-term and chronic insomnia (17). There has been no research on the volume of the basal forebrain in TLE until now. We found no differences in BF volume among the three groups. This could be because TLE originates mostly in the hippocampus and amygdala and has less direct influence on the BF or because differences in volume take a longer time to manifest. The small sample size of this study is another possible explanation for our findings. More information about changes in basal forebrain volume could be achieved from further studies with larger sample sizes and longitudinal designs.

### Changes in Functional Connectivity With the BF

The BF nuclei, which contain four distinct cell groups (Ch1–4), are the primary sources of cholinergic projections to the neocortex, amygdala, and hippocampus (31). In comparison to the non-FBTCS group, we discovered that FC was reduced between Ch1-3 and the bilateral striatum as well as the left posterior cerebellar lobe (PCL) in FBTCS patients; additionally, FC was reduced between Ch4 and the amygdala. The striatum is crucial for a number of complex functions, ranging from motor control to action selection and attention (32). Electron microscopy demonstrated that the ventral and dorsal striatum provide synaptic input to cholinergic BF neurons (33). A neuropathological analysis established a direct anatomical relationship between the striatum and basal forebrain, providing behavioral and structural evidence (34). Striatal structure and function are altered in attention-deficit/hyperactivity disorder (35) and a variety of epileptic conditions, including focal to bilateral tonic-clonic seizures (36) and pediatric epilepsy (37). Additionally, we found a significant correlation between the rsFC of Ch1-3 and the striatum and vigilance measurements. We postulate that recurrent seizures have a detrimental effect on the striatum, reducing ACh levels in the basal forebrain and impairing vigilance. Numerous studies have demonstrated that the cerebellum is essential for normal cognitive function. A lesion in the PCL can result in cerebellar cognitive-affective syndrome, characterized by issues with executive function, visual-spatial processing, linguistic abilities, and emotional regulation. In TLE with or without FBTCS as well as right TLE, we previously described aberrant FC between deep cerebellar nuclei and the cerebral cortex (38, 39). In the current study, we found decreased rsFC between Ch1-3 and the left posterior cerebellar lobe in FBTCS patients, which is consistent with earlier findings. Our findings imply that disruption of the rsFC between the basal forebrain and cerebellum may contribute to cognitive impairment in TLE. Prior research has established that the amygdala regulates the prefrontal cortex (PFC) and hippocampus directly via cholinergic projections from the basal forebrain and subsequent acetylcholine release (40). According to Adam et al. (40), cholinergic projections from the basal forebrain moderate activity in the greater amygdala during the processing of physiologically relevant stimuli in humans. The Ch4 subregion has cholinergic projections to the neocortex and amygdala. In our investigation, we found that the rsFC between Ch 4 and the amygdala was considerably lower in the FBTCS group than in the non-FBTCS group. We postulate that repeated FBTCS seizures disturb the basal forebrain-amygdala circuit by reducing the amount of ACh projected from the basal forebrain to the amygdala. Together, aberrant rsFC between basal subregions and the striatum, cerebellum posterior lobe, and amygdala influence alertness and may be a critical neuroimaging biomarker for differentiating FBTCS and non-FBTCS patients.

The DMN is involved in working memory, emotions, cognitive performance, and epileptic activity. Mounting data suggest that disrupting the DMN may cause epileptic activity, cognitive dysfunction, and mental dysfunction in TLE patients (41). The precuneus and middle temporal gyrus (MTG) are key components of the DMN, as they participate in processes related to consciousness, self-reflection, visuospatial function, and cognition (42–44). Nair et al. (45) demonstrated that gamma oscillations were restricted to the BF and that BF gamma-band activity had a direct effect on a DMN hub in rats, implying that the BF may be an important target for DMN regulation. In our study, both groups of patients displayed significantly lower rsFC between the right Ch1-3 and right precuneus as well as left MTG in comparison to HCs, indicating decreased synchronous neuronal activity between the DMN and basal forebrain. We speculate that the DMN might be a target for epilepsy and cognitive control in the basal forebrain. Both the DMN and the basal forebrain subregions influence cognitive function, suggesting that disrupted rsFC from the basal forebrain to the DMN could impact cognition in TLE patients.

In addition, compared to HCs, both patient groups had lower rsFC between Ch1-3 and cortical regions, such as the left superior frontal gyrus (SFG) and left occipital middle gyrus, indicating reduced cholinergic innervation in these cortical regions. Damage to the SFG, which is a crucial component of the frontoparietal network, might cause vigilance deficits. The FC between Ch1-3 and the left SFG was considerably lower in the patient groups than in the HCs, implying that diminished ACh in the frontal cortex may contribute to cognitive impairment in TLE. The findings were in line with earlier research that showed disturbed rsFC in the frontal cortices, linking it to deficits in alertness (46) and executive function (47) in TLE. fMRI studies have demonstrated that the visual cortex is engaged and cerebral blood flow is elevated during attention-related tasks (48, 49). In rTLE patients, the functional activity of the superior occipital gyrus in the alertness-related network was higher than that in HCs (46). Julia Schumacher et al. also identified aberrant functional connectivity between the basal forebrain and occipital cortex in Lewy body dementia and Alzheimer's disease, which they believe may indicate a cholinergic system imbalance and a shift in the cholinergic input to the occipital cortex (22). Additionally, gray matter volume reduction and hypometabolism were detected in the visual cortex of patients with TLE (50, 51). Therefore, our findings support the concept that in TLE the basal forebrain improperly modulates the frontoparietal and sensory networks, resulting in cognitive dysfunction.

### Limitations

This investigation has some limitations. First, the sample size is relatively small. As a result, our findings need to be confirmed in a broader patient group. Second, this was a cross-sectional study. Therefore, a longitudinal assessment of resting-state fMRI in temporal lobe epilepsy will be required to confirm these findings. Third, we did not take into account the effect of antiepileptic medicines on FC and VBM. Fourth, we cannot rule out the effect of interictal discharges on patient alertness because a synchronous electroencephalogram was not performed during the acquisition of imaging data. Finally, we defined BF subregions using a probabilistic map extracted from the SPM Anatomy Toolbox, which has been extensively used in previous research (1, 17). However, the BF subregions are segregated in additional ways (52). In the future, it is vital to employ different parcellation methods to acquire a thorough understanding of BF alterations in TLE.

## Conclusion

To the best of our knowledge, this is the first study to use BF subregions as seeds for performing FC analysis on patients with and without FBTCS. Patients with FBTCS showed disrupted rsFC between BF subregions and many brain regions compared to individuals without FBTCS or HCs. There was a substantial correlation between abnormal rsFC from the BF to the striatum and alertness metrics. Our findings reveal a link between altered basal forebrain-cerebral connections and reduced alertness in patients with FBTCS and suggest that cholinergic BF degradation may be a critical physiopathological mechanism underlying impaired alertness in TLE. Our results suggest that the BF subregions could serve as critical nodes for identifying TLE subtype-specific diagnostic and classification biomarkers, as well as more effective treatment alternatives.

## Data Availability Statement

The original contributions presented in the study are included in the article/Supplementary Material, further inquiries can be directed to the corresponding author/s.

## Ethics Statement

The studies involving human participants were reviewed and approved by Ethics Committee of The First Affiliated Hospital of Guangxi Medical University. The patients/participants provided their written informed consent to participate in this study.

## Author Contributions

BF and LP designed the study. BF conducted the study and data analyses. BF and SL wrote the manuscript. XZ handled the data curation and methodology. ZL and ZC oversaw data curation and investigation. JZ handled the funding acquisition and project administration. All authors finally agreed to publish this manuscript.

## Funding

This research was funded by the National Natural Science Foundation of China (Grant No. 81560223) and Natural Science Foundation of Guangxi Province (2016GXNSFAA380182).

## Conflict of Interest

The authors declare that the research was conducted in the absence of any commercial or financial relationships that could be construed as a potential conflict of interest.

## Publisher's Note

All claims expressed in this article are solely those of the authors and do not necessarily represent those of their affiliated organizations, or those of the publisher, the editors and the reviewers. Any product that may be evaluated in this article, or claim that may be made by its manufacturer, is not guaranteed or endorsed by the publisher.

## References

[1] González HF Narasimhan S Johnson GW Wills KE Haas KF Konrad PE. Role of the nucleus basalis as a key network node in temporal lobe epilepsy. Neurology. (2021) 96:e1334–46. 10.1212/wnl.0000000000011523 33441453 PMC8055321

[2] Fisher RS Cross JH French JA Higurashi N Hirsch E Jansen FE. Operational classification of seizure types by the international league against epilepsy: position paper of the ILAE commission for classification and terminology. Epilepsia. (2017) 58:522–30. 10.1111/epi.13670 28276060

[3] Caciagli L Allen LA He X Trimmel K Vos SB Centeno M. Thalamus and focal to bilateral seizures: a multiscale cognitive imaging study. Neurology. (2020) 95:e2427–41. 10.1212/WNL.0000000000010645 32847951 PMC7682917

[4] Sinha N Peternell N Schroeder GM de Tisi J Vos SB Winston GP. Focal to bilateral tonic-clonic seizures are associated with widespread network abnormality in temporal lobe epilepsy. Epilepsia. (2021) 62:729–41. 10.1111/epi.16819 33476430 PMC8600951

[5] Allone C Buono VL Corallo F Pisani LR Pollicino P Bramanti P. Neuroimaging and cognitive functions in temporal lobe epilepsy: a review of the literature. J Neurol Sci. (2017) 381:7–15. 10.1016/j.jns.2017.08.007 28991719

[6] Ives-Deliperi V Butler JT. Mechanisms of cognitive impairment in temporal lobe epilepsy: a systematic review of resting-state functional connectivity studies. Epilepsy Behav. (2021) 115:107686. 10.1016/j.yebeh.2020.107686 33360743

[7] Prevey ML Delaney RC Cramer JA Mattson RH Study VE. Complex partial and secondarily generalized seizure patients: cognitive functioning prior to treatment with antiepileptic medication. VA epilepsy cooperative study 264 group. Epilepsy Res. (1998) 30:1–9. 10.1016/s0920-1211(97)00091-0 9551840

[8] Liu J Zhang Z Zhou X Pang X Liang X Huang H. Disrupted alertness and related functional connectivity in patients with focal impaired awareness seizures in temporal lobe epilepsy. Epilepsy Behav. (2020) 112:107369. 10.1016/j.yebeh.2020.107369 32858367

[9] Posner MI. Measuring alertness. Ann NY Acad Sci. (2008) 1129:193–9. 10.1196/annals.1417.011 18591480

[10] Englot DJ D'Haese PF Konrad PE Jacobs ML Gore JC Abou-Khalil BW. Functional connectivity disturbances of the ascending reticular activating system in temporal lobe epilepsy. J Neurol Neurosurg Psych. (2017) 88:925–32. 10.1136/jnnp-2017-315732 28630376 PMC5634927

[11] Englot DJ Modi B Mishra AM DeSalvo M Hyder F Blumenfeld H. Cortical deactivation induced by subcortical network dysfunction in limbic seizures. J Neurosci. (2009) 29:13006–18. 10.1523/jneurosci.3846-09.2009 19828814 PMC2778759

[12] Blumenfeld H. Impaired consciousness in epilepsy. Lancet Neurol. (2012) 11:814–26. 10.1016/s1474-4422(12)70188-6 22898735 PMC3732214

[13] Zaborszky L Hoemke L Mohlberg H Schleicher A Amunts K Zilles K. Stereotaxic probabilistic maps of the magnocellular cell groups in human basal forebrain. Neuroimage. (2008) 42:1127–41. 10.1016/j.neuroimage.2008.05.055 18585468 PMC2577158

[14] Chen XM Huang DH Chen ZR Ye W Lv ZX Zheng JO. Temporal lobe epilepsy: decreased thalamic resting-state functional connectivity and their relationships with alertness performance. Epilepsy Behav. (2015) 44:47–54. 10.1016/j.yebeh.2014.12.013 25622022

[15] Gersel Stokholm M Iranzo A Østergaard K Serradell M Otto M Bacher Svendsen K. Cholinergic denervation in patients with idiopathic rapid eye movement sleep behaviour disorder. Eur J Neurol. (2020) 27:644–52. 10.1111/ene.14127 31725927

[16] Kato S Watanabe H Senda J Hirayama M Ito M Atsuta N. Widespread cortical and subcortical brain atrophy in Parkinson's disease with excessive daytime sleepiness. J Neurol. (2012) 259:318–26. 10.1007/s00415-011-6187-6 21850388

[17] Ma X Fu S Yin Y Wu Y Wang T Xu G. Aberrant functional connectivity of basal forebrain subregions with cholinergic system in short-term and chronic insomnia disorder. J Affect Disord. (2021) 278:481–7. 10.1016/j.jad.2020.09.103 33011526

[18] Grossberg S. Acetylcholine neuromodulation in normal and abnormal learning and memory: vigilance control in waking, sleep, autism, amnesia and alzheimer's disease. Front Neural Circuits. (2017) 11:82. 10.3389/fncir.2017.00082 29163063 PMC5673653

[19] Mesulam MM Geula C. Nucleus basalis (Ch4) and cortical cholinergic innervation in the human brain: observations based on the distribution of acetylcholinesterase and choline acetyltransferase. J Comp Neurol. (1988) 275:216–40. 10.1002/cne.902750205 3220975

[20] Mesulam MM Mufson EJ Levey AI Wainer BH. Cholinergic innervation of cortex by the basal forebrain: cytochemistry and cortical connections of the septal area, diagonal band nuclei, nucleus basalis (substantia innominata), and hypothalamus in the rhesus monkey. J Comp Neurol. (1983) 214:170–97. 10.1002/cne.902140206 6841683

[21] Ballinger EC Ananth M Talmage DA Role LW. Basal forebrain cholinergic circuits and signaling in cognition and cognitive decline. Neuron. (2016) 91:1199–218. 10.1016/j.neuron.2016.09.006 27657448 PMC5036520

[22] Schumacher J Thomas AJ Peraza LR Firbank M O'Brien JT Taylor JP. Functional connectivity of the nucleus basalis of Meynert in Lewy body dementia and Alzheimer's disease. Int Psychoger IF 2.4. (2021) 33:89–94. 10.1017/s1041610220003944 33413710 PMC8482375

[23] Wilson H de Natale ER Politis M. Nucleus basalis of Meynert degeneration predicts cognitive impairment in Parkinson's disease. Handb Clin Neurol. (2021) 179:189–205. 10.1016/b978-0-12-819975-6.00010-8 34225962

[24] Wu Y Hu S Wang Y Dong T Wu H Zhang Y. The degeneration changes of basal forebrain are associated with prospective memory impairment in patients with Wilson's disease. Brain Behav. (2021) 11:e2239. 10.1002/brb3.2239 34124853 PMC8413803

[25] Hildesheim FE Benedict RH Zivadinov R Dwyer MG Fuchs T Jakimovski D. Weinstock-Guttman, and N. Bergsland. Nucleus basalis of meynert damage and cognition in patients with multiple sclerosis. J Neurol. (2021) 268:4796–808. 10.1007/s00415-021-10594-7 33997915 PMC8568637

[26] Scheffer IE Berkovic S Capovilla G Connolly MB French J Guilhoto L. ILAE classification of the epilepsies: position paper of the ILAE commission for classification and terminology. Epilepsia. (2017) 58:512–21. 10.1111/epi.13709 28276062 PMC5386840

[27] Fan J Gu X Guise KG Liu X Fossella J Wang H. Testing the behavioral interaction and integration of attentional networks. Brain Cogn. (2009) 70:209–20. 10.1016/j.bandc.2009.02.002 19269079 PMC2674119

[28] Schrouff J Rosa MJ Rondina JM Marquand AF Chu C Ashburner J. PRoNTo: pattern recognition for neuroimaging toolbox. Neuroinformatics. (2013) 11:319–37. 10.1007/s12021-013-9178-1 23417655 PMC3722452

[29] Schumacher J Taylor JP Hamilton CA Firbank M Cromarty RA Donaghy PC. *In vivo* nucleus basalis of Meynert degeneration in mild cognitive impairment with Lewy bodies. Neuroimage Clin. (2021) 30:102604. 10.1016/j.nicl.2021.102604 33711623 PMC7972982

[30] Wilkins KB Parker JE Bronte-Stewart HM. Gait variability is linked to the atrophy of the nucleus basalis of Meynert and is resistant to STN DBS in Parkinson's disease. Neurobiol Dis. (2020) 146:105134. 10.1016/j.nbd.2020.105134 33045357 PMC7711311

[31] Woolf NJ. Cholinergic systems in mammalian brain and spinal cord. Prog Neurobiol. (1991) 37:475–524. 10.1016/0301-0082(91)90006-m 1763188

[32] Valjent E Gangarossa G. The Tail of the striatum: from anatomy to connectivity and function. Trends Neurosci. (2021) 44:203–14. 10.1016/j.tins.2020.10.016 33243489

[33] Záborszky L Gombkoto P Varsanyi P Gielow MR Poe G Role LW. Specific basal forebrain–cortical cholinergic circuits coordinate cognitive operations. J Neurosci: Off J Soc Neurosci. (2018) 38:9446–58. 10.1523/JNEUROSCI.1676-18.2018 30381436 PMC6209837

[34] Shu SY Jiang G Zheng Z Ma L Wang B Zeng Q. A new neural pathway from the ventral striatum to the nucleus basalis of meynert with functional implication to learning and memory. Mol Neurobiol. (2019) 56:7222–33. 10.1007/s12035-019-1588-0 31001802 PMC6728281

[35] Greven CU Bralten J Mennes M O'Dwyer L van Hulzen KJ Rommelse N. Developmentally stable whole-brain volume reductions and developmentally sensitive caudate and putamen volume alterations in those with attention-deficit/hyperactivity disorder and their unaffected siblings. JAMA Psychiatry. (2015) 72:490–9. 10.1001/jamapsychiatry.2014.3162 25785435

[36] Xu Q Zhang Q Yang F Weng Y Xie X Hao J. Stufflebeam,. Cortico-striato-thalamo-cerebellar networks of structural covariance underlying different epilepsy syndromes associated with generalized tonic-clonic seizures. Hum Brain Mapp. (2021) 42:1102–15. 10.1002/hbm.25279 33372704 PMC7856655

[37] MacEachern SJ Santoro JD Hahn KJ Medress ZA Stecher X Li MD. Children with epilepsy demonstrate macro- and microstructural changes in the thalamus, putamen, and amygdala. Neuroradiology. (2020) 62:389–97. 10.1007/s00234-019-02332-8 31853588

[38] Nie L Jiang Y Lv Z Pang X Liang X Chang W. Deep cerebellar nuclei functional connectivity with cerebral cortex in temporal lobe epilepsy with and without focal to bilateral tonic-clonic seizures: a resting-state fMRI study. Cerebellum. (2022) 21:253–63. 10.1007/s12311-021-01266-3 34164777

[39] Zhou X Zhang Z Liu J Qin L Pang X Zheng J. Disruption and lateralization of cerebellar-cerebral functional networks in right temporal lobe epilepsy: a resting-state fMRI study. Epilepsy Behav. (2019) 96:80–6. 10.1016/j.yebeh.2019.03.020 31103016

[40] Gorka AX Knodt AR Hariri AR. Basal forebrain moderates the magnitude of task-dependent amygdala functional connectivity. Soc Cogn Affect Neurosci. (2015) 10:501–7. 10.1093/scan/nsu080 24847112 PMC4381234

[41] Gonen OM Kwan P O'Brien TJ Lui E. Resting-state functional MRI of the default mode network in epilepsy. Epilepsy Behav. (2020) 111:107308. 10.1016/j.yebeh.2020.107308 32698105

[42] Cunningham SI Tomasi D Volkow ND. Structural and functional connectivity of the precuneus and thalamus to the default mode network. Hum Brain Mapp. (2017) 38:938–56. 10.1002/hbm.23429 27739612 PMC6866740

[43] Cavanna AE. The precuneus and consciousness. CNS Spectr. (2007) 12:545–52. 10.1017/s1092852900021295 17603406

[44] Mahayana IT Tcheang L Chen CY Juan CH Muggleton NG. The precuneus and visuospatial attention in near and far space: a transcranial magnetic stimulation study. Brain Stimul. (2014) 7:673–9. 10.1016/j.brs.2014.06.012 25112521

[45] Nair J Klaassen AL Arato J Vyssotski AL Harvey M Rainer G. Basal forebrain contributes to default mode network regulation. Proc Natl Acad Sci USA. (2018) 115:1352–7. 10.1073/pnas.1712431115 29363595 PMC5819396

[46] Li J Chen X Ye W Jiang W Liu H Zheng J. Alteration of the alertness-related network in patients with right temporal lobe epilepsy: a resting state fMRI study. Epilepsy Res. (2016) 127:252–9. 10.1016/j.eplepsyres.2016.09.013 27661438

[47] Zhou X Zhang Z Liu J Qin L Zheng J. Aberrant topological organization of the default mode network in temporal lobe epilepsy revealed by graph-theoretical analysis. Neurosci Lett. (2019) 708:134351. 10.1016/j.neulet.2019.134351 31247225

[48] Kim J Whyte J Wang J Rao H Tang KZ Detre JA. Continuous ASL perfusion fMRI investigation of higher cognition: quantification of tonic CBF changes during sustained attention and working memory tasks. Neuroimage. (2006) 31:376–85. 10.1016/j.neuroimage.2005.11.035 16427324 PMC2362398

[49] Prado J Weissman DH. Weissman. Spatial attention influences trial-by-trial relationships between response time and functional connectivity in the visual cortex. Neuroimage. (2011) 54:465–73. 10.1016/j.neuroimage.2010.08.038 20736069

[50] Akman CI Ichise M Olsavsky A Tikofsky RS Van Heertum RL Gilliam F. Epilepsy duration impacts on brain glucose metabolism in temporal lobe epilepsy: results of voxel-based mapping. Epilepsy Behav. (2010) 17:373–80. 10.1016/j.yebeh.2009.12.007 20149754 PMC10694865

[51] Kim JS Koo DL Joo EY Kim ST Seo DW Hong SB. Asymmetric gray matter volume changes associated with epilepsy duration and seizure frequency in temporal-lobe-epilepsy patients with favorable surgical outcome. J Clin Neurol. (2016) 12:323–31. 10.3988/jcn.2016.12.3.323 27449913 PMC4960217

[52] Herdick M Dyrba M Fritz HC Altenstein S Ballarini T Brosseron F. Multimodal MRI analysis of basal forebrain structure and function across the Alzheimer's disease spectrum. Neuroimage Clin. (2020) 28:102495. 10.1016/j.nicl.2020.102495 33395986 PMC7689403

