# Supplementary material for: Correlation Between the Functional Connectivity of Basal Forebrain Subregions and Vigilance Dysfunction in Temporal Lobe Epilepsy With and Without Focal to Bilateral Tonic-Clonic Seizure
Source: Front Psychiatry. 2022 Jun 2;13:888150. doi: 10.3389/fpsyt.2022.888150 (PMC9201520; doi:10.3389/fpsyt.2022.888150)

In our experiment, we explored whether there were differences in the brain regions between left and right mTLE in the different TLE groups with an ANOVA. We found that there was no significant difference (P＞0.05) between the two TLE groups. Details are shown in Figure 1 and Figure 2.

Fig 1


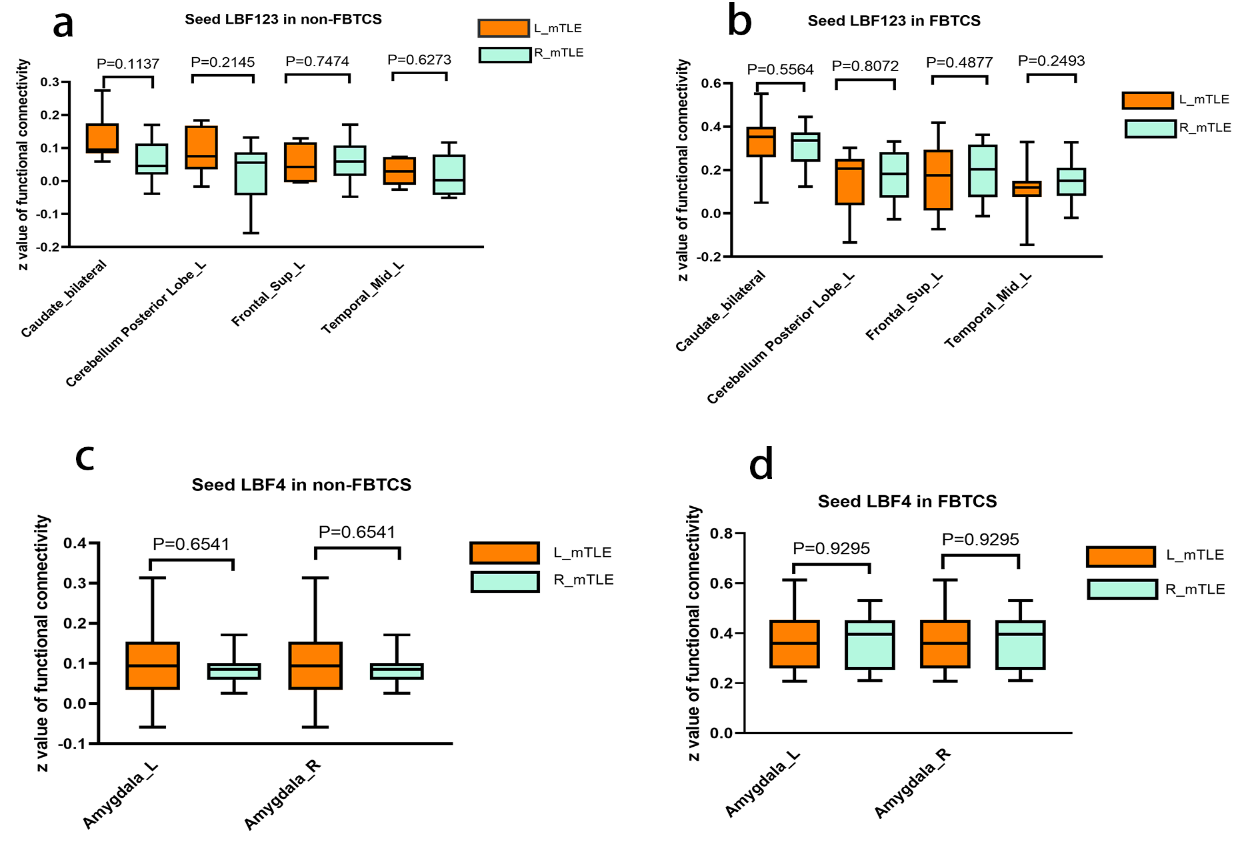


Fig 2


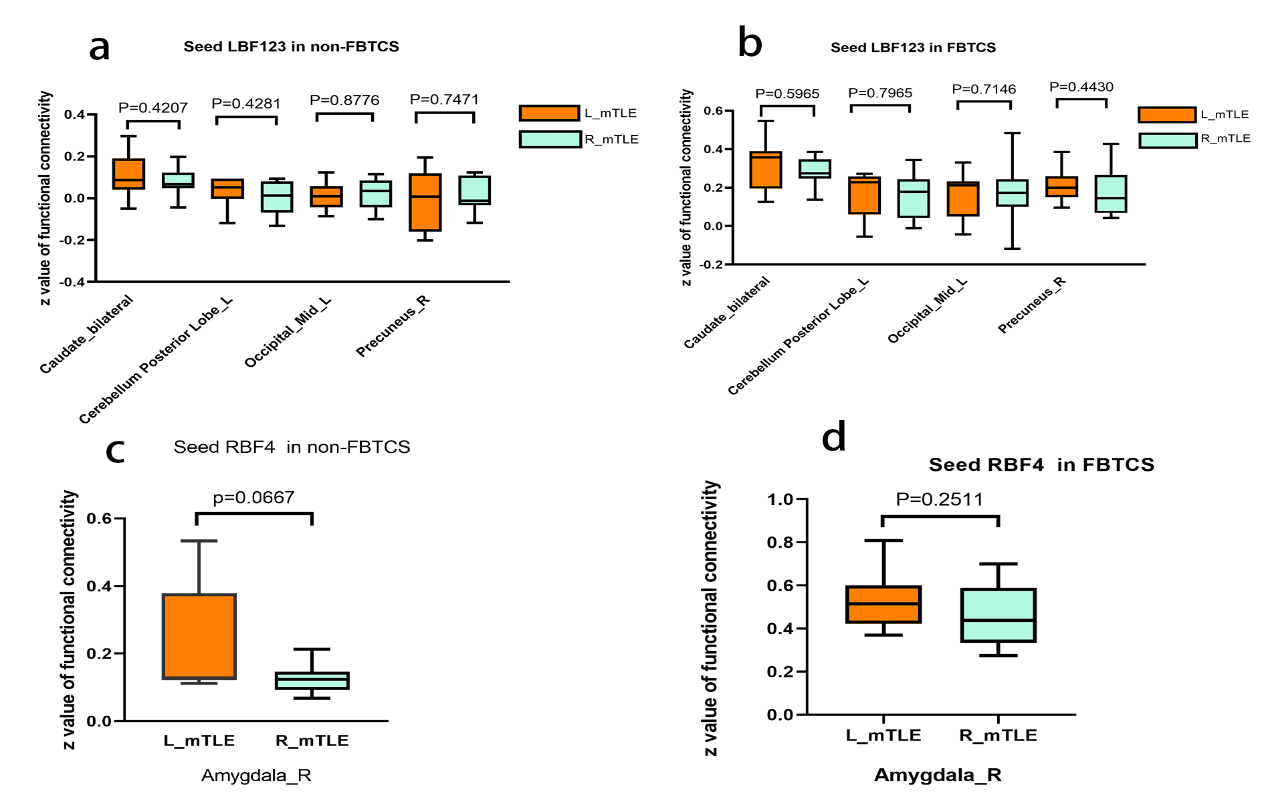

Supplement: Supplementary file 1 [file Table_1.DOCX]
